# Pasireotide can induce sustained decreases in urinary cortisol and provide clinical benefit in patients with Cushing’s disease: results from an open-ended, open-label extension trial

**DOI:** 10.1007/s11102-014-0618-1

**Published:** 2014-12-24

**Authors:** Jochen Schopohl, Feng Gu, Robert Rubens, Luc Van Gaal, Jérôme Bertherat, Monica Ligueros-Saylan, Andrew Trovato, Gareth Hughes, Luiz R. Salgado, Marco Boscaro, Rosario Pivonello

**Affiliations:** 1 Medizinische Klinik IV, University of Munich, Munich, Germany; 2 Department of Endocrinology, Key Laboratory of Endocrinology, Ministry of Health, Peking Union Medical College Hospital, Beijing, China; 3 Department of Endocrinology and Metabolism, University Hospital Ghent, Ghent, Belgium; 4 Department of Endocrinology, Diabetology and Metabolism, Antwerp University Hospital, Edegem (Antwerp), Belgium; 5 INSERM U1016, Institut Cochin, Paris, France; 6 Reference Centre for Rare Adrenal Diseases, Department of Endocrinology, Assistance Publique Hôpitaux de Paris, Hôpital Cochin, Paris, France; 7 University Paris Descartes, Paris, France; 8 Novartis Pharmaceuticals Corporation, East Hanover, NJ USA; 9 Novartis Pharma AG, Basel, Switzerland; 10 Division of General Internal Medicine, Hospital das Clínicas, University of São Paulo Medical School, São Paulo, Brazil; 11 Endocrinology Unit, Department of Medicine (DIMED), University of Padova, Padua, Italy; 12 Dipartimento di Medicina Clinica e Chirurgia, Università Federico II di Napoli, Via Sergio Pansini 5, 80131 Naples, Italy

**Keywords:** Pasireotide, Cushing’s disease, Somatostatin analogue, Corticotroph tumor, Urinary free cortisol

## Abstract

**Purpose:**

Report the efficacy and safety of pasireotide sc in patients with Cushing’s disease during an open-ended, open-label extension to a randomized, double-blind, 12-month, Phase III study.

**Methods:**

162 patients entered the core study. 58 patients who had mean UFC ≤ ULN at month 12 or were benefiting clinically from pasireotide entered the extension. Patients received the same dose of pasireotide as at the end of the core study (300–1,200 μg bid). Dose titration was permitted according to efficacy or drug-related adverse events.

**Results:**

40 patients completed 24 months’ treatment. Of the patients who entered the extension, 50.0 % (29/58) and 34.5 % (20/58) had controlled UFC (UFC ≤ ULN) at months 12 and 24, respectively. The mean percentage decrease in UFC was 57.3 % (95 % CI 40.7–73.9; n = 52) and 62.1 % (50.8–73.5; n = 33) after 12 and 24 months’ treatment, respectively. Improvements in clinical signs of Cushing’s disease were sustained up to month 24. The most frequent drug-related adverse events in patients who received ≥1 dose of pasireotide (n = 162) from core baseline until the 24-month cut-off were diarrhea (55.6 %), nausea (48.1 %), hyperglycemia (38.9 %), and cholelithiasis (31.5 %). No new safety issues were identified during the extension.

**Conclusions:**

Reductions in mean UFC and improvements in clinical signs of Cushing’s disease were maintained over 24 months of pasireotide treatment. The safety profile of pasireotide is typical for a somatostatin analogue, except for the frequency and degree of hyperglycemia; patients should be monitored for changes in glucose homeostasis. Pasireotide represents the first approved pituitary-targeted treatment for patients with Cushing’s disease.

**Electronic supplementary material:**

The online version of this article (doi:10.1007/s11102-014-0618-1) contains supplementary material, which is available to authorized users.

## Introduction

Cushing’s disease is a rare and debilitating disorder of hypercortisolism caused by an adrenocorticotropic hormone (ACTH)-secreting pituitary tumor [1–3]. Cushing’s disease is associated with increased morbidity and mortality due to the development of several hypercortisolism-induced clinical complications, including central obesity, insulin resistance, glucose intolerance, diabetes mellitus, hypertension and cardiovascular disease, as well as osteoporosis, psychiatric disorders and increased susceptibility to infection [1, 4, 5]. Transsphenoidal adenomectomy is the first-line therapy for most patients with Cushing’s disease. Second-line treatment options include repeat surgery, radiotherapy, bilateral adrenalectomy and medical therapy. Pasireotide (Signifor^®^) is a multireceptor-targeted somatostatin analogue with a high affinity for somatostatin receptor subtype 5 (sst_5_), as well as activating sst_1,2,3_ [6]. Pasireotide targets the underlying cause of Cushing’s disease by activating sst_5_ on corticotroph adenomas, resulting in the inhibition of ACTH secretion [7, 8].

The efficacy and safety of pasireotide has recently been evaluated in the largest prospective study of a medical therapy in Cushing’s disease [9]. This randomized, Phase III study led to the approval of pasireotide in the European Union, the United States of America and other countries worldwide for use in adult patients with Cushing’s disease for whom surgery is not an option or has failed [10, 11]. 12 months’ treatment with pasireotide was associated with a rapid and sustained reduction in urinary free cortisol (UFC) and significant improvements in the clinical signs and symptoms of Cushing’s disease [9]. The safety profile of pasireotide in this study was similar to that of other somatostatin analogues, except for the increased frequency and degree of hyperglycemia. Reported here are the safety and efficacy results for an additional 12 months of pasireotide treatment in a planned, open-label extension to this Phase III study.

## Methods

### Participant eligibility criteria

This open-label study was a planned extension to a 12-month, multicenter, randomized, Phase III study (Clinicaltrials.gov, NCT00434148) [9]. Patients who were eligible for entry into the core study were adults (≥18 years of age) with confirmed de novo, persistent or recurrent Cushing’s disease. Cushing’s disease was defined by a mean 24-h UFC level (calculated from four UFC samples collected within 14 days) ≥1.5 times the upper limit of normal (ULN; 145 nmol/24 h [52.5 μg/24 h]), a morning plasma ACTH level ≥5 ng/L (1.1 nmol/L), and a confirmed pituitary source of Cushing’s syndrome. Key exclusion criteria were tumor compression of the optic chiasm causing visual-field defects, pituitary irradiation within the previous 10 years, mitotane treatment within the previous 6 months, symptomatic cholelithiasis, a glycated hemoglobin (HbA_1c_) level >8 %, and de novo Cushing’s disease in patients who were surgical candidates. Patients with a mean UFC level ≤ ULN at month 12 or those considered, at the discretion of the investigator, to be achieving significant clinical benefit from pasireotide were eligible for entry into the extension.

The study was approved by the independent ethics committee, research ethics board or institutional review board at each center and complied with the ICH Harmonized Tripartite Guidelines for Good Clinical Practice, the Declaration of Helsinki and local laws. All patients provided written informed consent. All researchers had full access to all of the data in the study.

### Study design

At the beginning of the core study (i.e., core baseline), 162 patients were randomly assigned to receive subcutaneous (sc) pasireotide at a dose of 600 µg (n = 82) or 900 µg (n = 80) twice daily (bid). Patients with a mean UFC level ≤2 × ULN and not exceeding the baseline level at month 3 continued to receive their randomly assigned dose; all others had a dose increase of 300 μg bid. At month 6, patients entered an open-label phase until month 12; during this phase, dose increases of 300 µg bid were permitted in patients with UFC > ULN. Patients entering the open-ended extension continued, without interruption to treatment, on the same dose of pasireotide they were receiving at the end of the 12-month core study. During the extension, the dose could be increased by 300 µg bid at any time if UFC was higher than the ULN. The maximum permitted dose of pasireotide was 1,200 µg bid. In the event of drug-related adverse events (AEs), dose reductions of 300 µg bid were allowed.

### Efficacy assessments

During the extension, UFC, fasting serum cortisol and plasma ACTH levels were measured at 3-month intervals. The UFC level was calculated as the mean value from two consecutive 24-h urine samples; patients were considered to have controlled (UFC ≤ ULN), partially controlled (UFC > ULN but ≥50 % decrease from core baseline), or uncontrolled (UFC > ULN without a ≥50 % decrease from core baseline) UFC. Patients with a missing mean UFC value were considered to be uncontrolled at the corresponding time point; patients who discontinued treatment were considered to be uncontrolled at all subsequent time points.

### Assay details

UFC level was determined by high-performance liquid chromatography [Alliance^®^ 2795 High Throughput System, Waters Corp, Milford, MA, USA; normal UFC range 30–145 nmol/24 h (10.8–52.5 μg/24 h)], as described previously [12]. All samples were analyzed by central laboratories (Eurofins Medinet BV, Breda, The Netherlands; CRL Medinet Inc, Lenexa, KS, USA; and Eurofins Technology Services [Suzhou] Co Ltd, Suzhou, China). Pre-dose blood samples were tested for serum cortisol (assay: ADVIA Centaur^®^ CP Immunoassay System, Siemens Healthcare Diagnostics Inc, Tarrytown, NY, USA) and plasma ACTH (assay: Immulite^®^ 2000 ACTH kit, DPC, Los Angeles, CA, USA).

### Clinical signs and safety assessments

Systolic and diastolic blood pressure, body weight, body mass index (BMI), and total and low-density lipoprotein (LDL) cholesterol levels were assessed at 3-month intervals during the extension. At each visit, hematological and blood biochemical measurements (including fasting blood glucose and HbA_1c_ levels) and urinalysis were performed. Patient diabetic status was defined as normal glucose tolerance (HbA_1c_ < 5.7 %), pre-diabetes (HbA_1c_ ≥5.7 to <6.5 %), and diabetes (HbA_1c_ ≥ 6.5 %); patients with HbA_1c_ levels ≥8 % were considered to have poorly controlled diabetes. AEs were graded according to Common Terminology Criteria for Adverse Events (CTCAE) version 3.0 [13]. All medications, other than study drug, administered during the study were recorded; however, dose information for concomitant medications was not recorded during the study.

### Statistical analysis

Analysis of mean UFC, serum cortisol, plasma ACTH, and clinical signs was performed for those patients who had evaluable measurements at the specific time point; for calculations of mean change, only those patients who had evaluable measurements at both core baseline and the specific time point were included. All randomized patients who received at least one dose of pasireotide (n = 162) were included in the analysis of safety and AEs, unless otherwise stated. The 24-month data cut-off for AE analysis occurred when the last ongoing patient reached 24 months’ pasireotide treatment (25 March 2011). As such, a number of patients included in the safety analysis received >24 months of pasireotide treatment. The study was not powered to detect statistically significant differences between dose groups, and these comparisons were not performed.

## Results

### Patients

Seventy-eight of the 162 patients (48.1 %) who entered the core study completed 12 months’ pasireotide treatment (39 patients each in both the 600 and 900 µg bid groups). Of these 78 patients, 58 entered the optional extension phase; the reasons why patients chose not to enter the extension were not consistently recorded. Demographics and characteristics for the 58 patients who entered the extension (26 and 32 patients in the 600 and 900 µg bid groups, respectively) are shown in Table 1. The mean total duration of treatment from core baseline up to the 24-month data cut-off for those all patients who received ≥1 dose of pasireotide (n = 162) was 14 months (range 0–50) and for those who entered the extension phase (n = 58) was 27 months (range 12–50); 53, 46, 41, and 40 patients completed 15, 18, 21, and 24 months’ treatment, respectively, while 10 patients received ≥36 months’ treatment. The mean daily dose of pasireotide for patients randomized to the 600 and 900 µg arms was 1,185 and 1,739 μg for months 0–6, 1,420 and 1,813 μg for months 6–12, 1,509 and 1,766 μg for months 12–18, and 1,500 and 1,620 μg for months 18–24.

**Table 1:** Summary of core baseline demographics and characteristics (by dose group) of patients who entered the open-ended extension

Characteristic	Pasireotide 600 µg bid (N = 26)	Pasireotide 900 µg bid (N = 32)	Overall (N = 58)
Females, n (%)	20 (77)	31 (97)	51 (88)
Age, years			
Mean (range)	40.4 (18–67)	41.4 (19–58)	40.9 (18–67)
≥65 years, n (%)	1 (4)	0 (0)	1 (2)
Time since diagnosis, months			
Mean (range)	62 (0.8–341.8)	57 (0.1–216.3)	59 (0.1–341.8)
Previous treatment, n (%)			
Surgery	20 (77)	23 (72)	43 (74)
Medication	10 (38)	17 (53)	27 (47)
Pituitary irradiation	3 (12)	3 (9)	6 (10)
Cushing’s disease status, n (%)			
De novo	5 (19)	6 (19)	11 (19)
Persistent/recurrent	21 (81)	26 (81)	47 (81)
UFC, nmol/24 h			
Baseline measurement, n*	24	29	53
Mean	842	529	671
Median (range)	764 (220–4,564)	448 (195–1,758)	511 (195–4,564)

* Baseline UFC was calculated if ≥3 samples were collected

### Efficacy

#### Urinary free cortisol

**Fig. 1:**
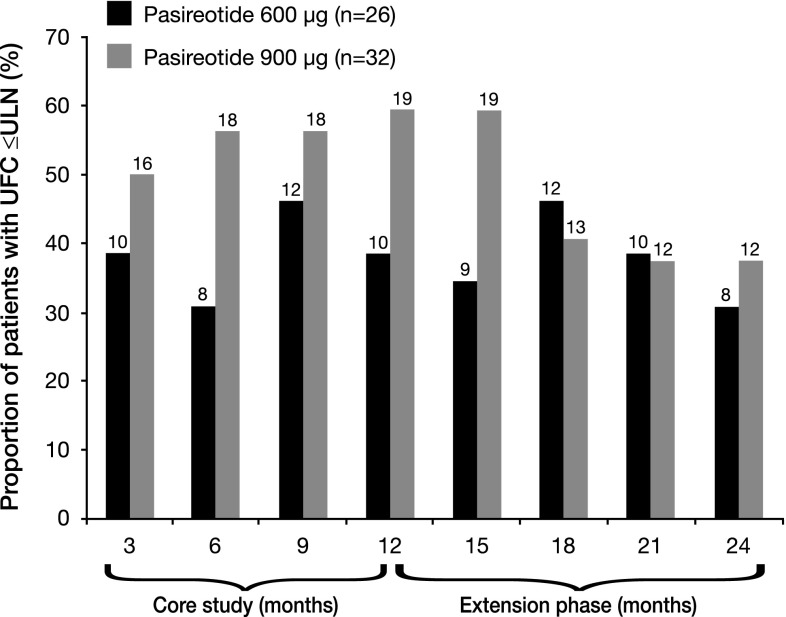
Proportion of patients with UFC ≤ ULN at time points up to month 24 in the 58 patients who entered the open-ended extension. The numbers shown above *each bar* represent the total number of patients in each treatment group with UFC ≤ ULN at the specific time point. Patients with missing data are classed as non-responders at the given time point; patients who discontinued are classed as non-responders at subsequent time points. The pasireotide 600 and 900 µg dose groups represent the randomly assigned treatment at core baseline; patients were not necessarily receiving their randomized dose at each time point

Among the 58 patients who entered the extension, controlled UFC (UFC ≤ ULN) was achieved at month 12 (end of the core study/extension baseline) by 50.0 % (29/58) of patients; 20.7 % (12/58) and 29.3 % (17/58) of patients had partially controlled UFC (UFC > ULN but ≥50 % decrease from core baseline) and uncontrolled UFC, respectively. During the core phase, the proportion of patients achieving UFC ≤ ULN was dose dependent; at month 12, 38.5 % (10/26) of patients in the 600 µg bid group and 59.4 % (19/32) of patients in the 900 µg bid group had controlled UFC (Fig. 1). This dose–response relationship was less apparent after 15 months of treatment; at month 24, 30.8 % (8/26) and 37.5 % (12/32) of patients in the 600 and 900 µg bid groups, respectively, had controlled UFC.

**Fig. 2:**
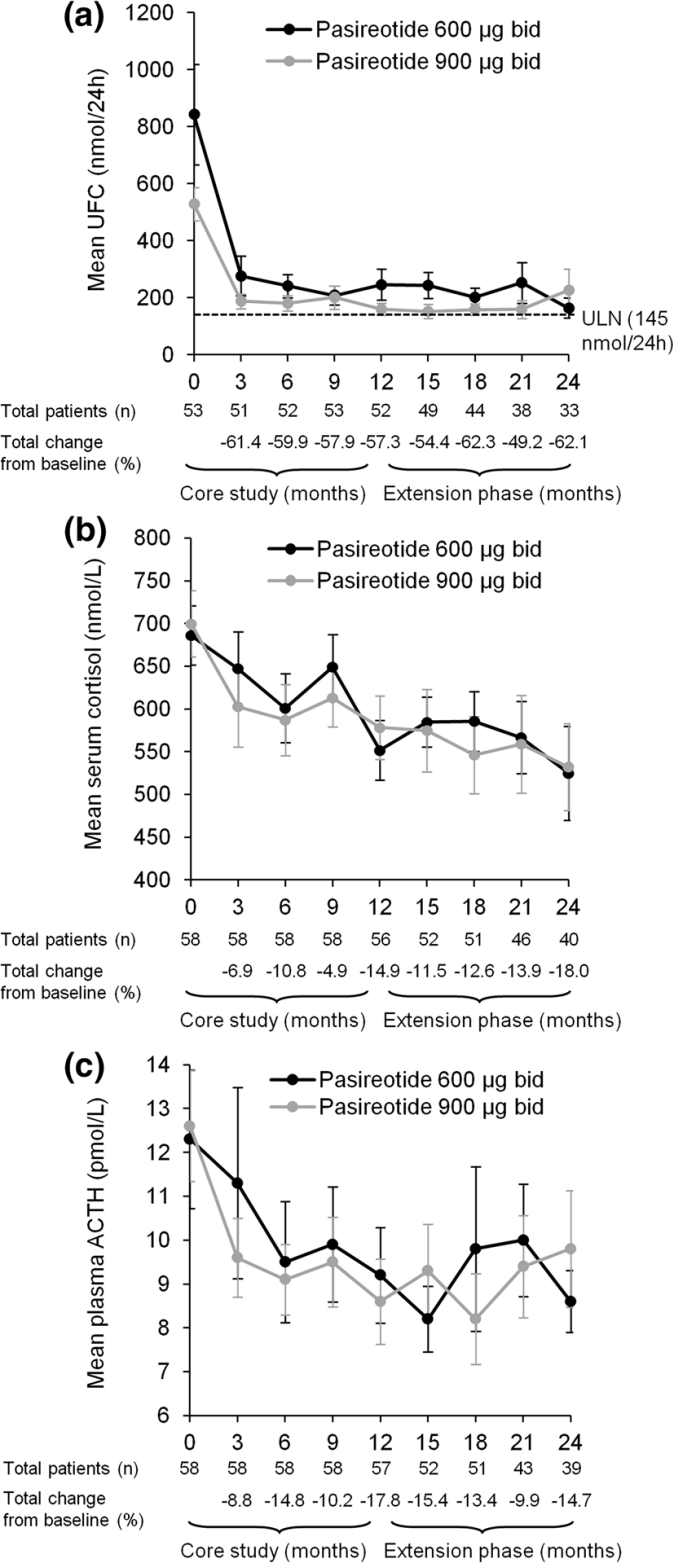
Mean (±SE) **a** UFC, **b** serum cortisol, and **c** plasma ACTH levels from baseline up to month 24 in the 58 patients who entered the open-ended extension. The total numbers of patients with evaluable measurements for UFC, serum cortisol, and plasma ACTH are shown beneath each graph

The mean percentage decrease in UFC level from core baseline during the first 12 months of treatment [57.3 % (95 % CI 40.7 to 73.9)] in patients who entered the extension phase (n = 58) was maintained throughout the 24-month study period (Fig. 2); the mean percentage decrease in UFC level from baseline to month 18 and month 24 was 62.3 % (53.4 to 71.1) and 62.1 % (50.8 to 73.5), respectively.

Of the 29 patients who had controlled UFC levels at the end of the core study, 48.3 % (14/29) and 10.3 % (3/29) had controlled or partially controlled UFC levels, respectively, at month 24, while 41.4 % (12/29) were uncontrolled. Six of the 12 patients who were classified as uncontrolled at month 24 discontinued treatment during the extension (reasons for discontinuation: AE, n = 2; consent withdrawal, n = 2; unsatisfactory therapeutic effect, n = 2); at last available assessment, two of these patients were controlled, two were partially controlled, and two were uncontrolled. One additional patient, who had controlled UFC at month 12 and at last available assessment, had a missing mean UFC value at month 24.

Of the 12 patients who had partially controlled UFC at month 12, 16.7 % (2/12) remained partially controlled, 25.0 % (3/12) had controlled UFC, and 58.3 % (7/12) had uncontrolled UFC levels at month 24. Three of the 17 (17.6 %) patients who were uncontrolled at month 12 achieved controlled UFC levels at month 24, while 14 (82.4 %) remained uncontrolled.

#### Serum cortisol and plasma ACTH

Sustained mean reductions in serum cortisol and plasma ACTH levels were observed throughout the 24-month treatment period (Fig. 2). The mean percentage decrease in morning serum cortisol level at months 12 and 24 was 14.9 % (95 % CI 7.8 to 22.0) and 18.0 % (95 % CI 5.8 to 30.2), respectively. The mean percentage decrease in plasma ACTH was 17.8 % (95 % CI 5.2 to 30.5) and 14.7 % (95 % CI 2.6 to 26.9) at months 12 and 24, respectively.

### Clinical signs

**Fig. 3:**
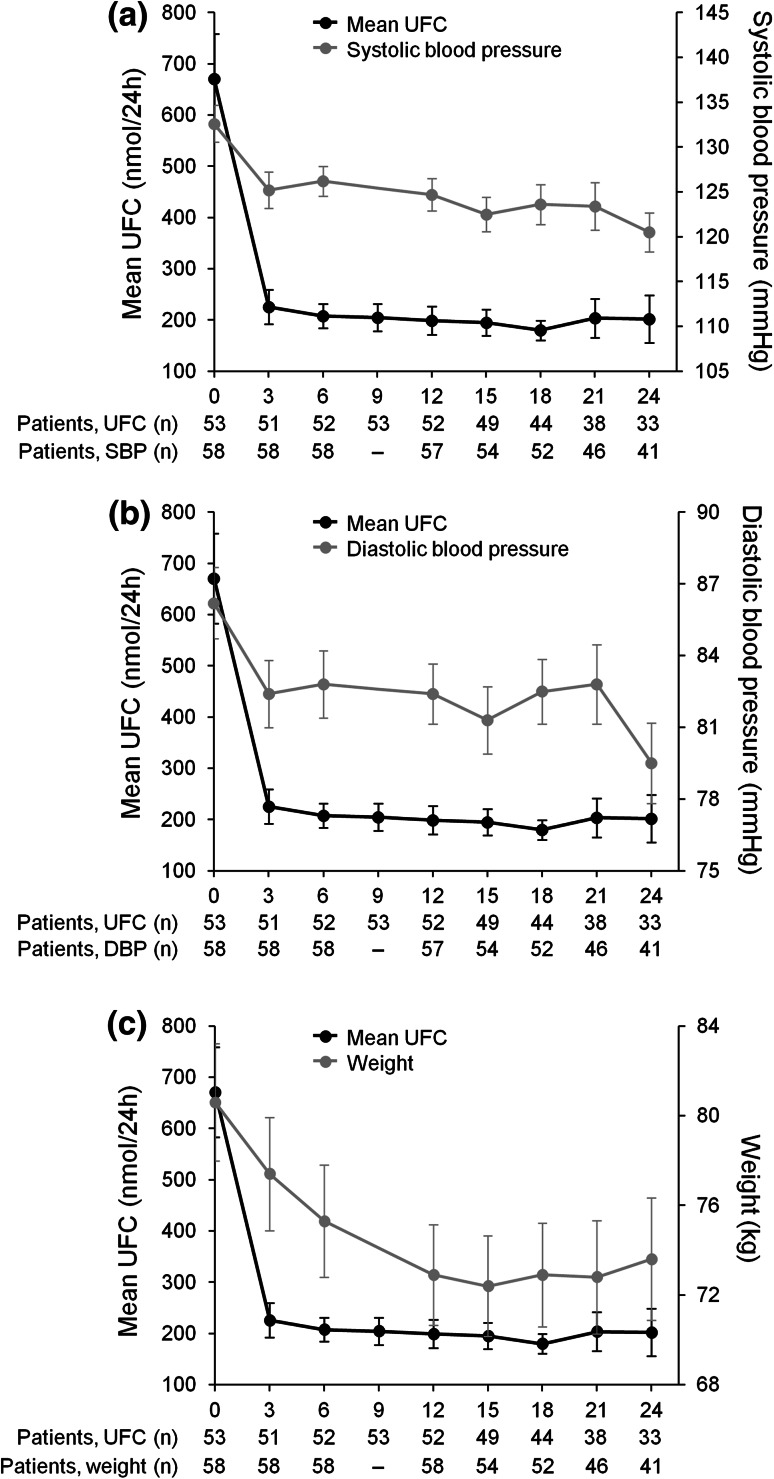
Mean UFC (±SE) and **a** systolic blood pressure, **b** diastolic blood pressure, and **c** weight up to month 24 in the 58 patients who entered the open-ended extension. The total numbers of patients included in the analyses of mean UFC level and mean signs and symptoms scores are shown beneath each graph. *DBP* diastolic blood pressure, *SBP* systolic blood pressure

Marked improvements in clinical signs of Cushing’s disease were observed during the 24-month study; these effects were observed by month 12 and were sustained until month 24 (Fig. 3). Mean changes from core baseline following 12 months of pasireotide treatment for those patients who entered the extension were: systolic blood pressure, −8.4 mmHg (95 % CI −12.3 to −4.5; −5.6 % change); diastolic blood pressure, −4.0 mmHg (95 % CI −6.9 to −1.2; −3.6 % change); weight, −7.7 kg (95 % CI −9.3 to −6.1; −9.1 % change); and BMI, −2.8 kg/m^2^ (95 % CI −3.4 to −2.3; −9.1 % change). Further improvements in several clinical signs were observed following extended pasireotide treatment; mean changes from core baseline at 24 months were: systolic blood pressure, −11.3 mmHg (95 % CI −15.0 to −7.5; −8.2 % change); diastolic blood pressure, −7.2 mmHg (95 % CI −10.4 to −3.9; −7.6 % change); weight, −8.7 kg (95 % CI −11.1 to −6.2; −9.6 % change); and BMI −3.2 kg/m^2^ (95 % CI −4.0 to −2.3; −9.6 % change). Mean changes from core baseline in total and LDL cholesterol levels were −0.6 mmol/L (95 % CI −0.8 to −0.3; −8.5 % change) and −0.4 mmol/L (95 % CI −0.7 to −0.2; −10.4 % change), respectively, at month 12 and −0.4 mmol/L (95 % CI −0.8 to −0.1; −5.8 % change) and −0.3 mmol/L (95 % CI −0.6 to 0.0; −3.0 % change), respectively, at month 24.

### Safety and tolerability

Of the patients who entered the open-ended extension, 46.6 % (27/58) had discontinued the study prior to the 24-month data cut-off: 12 because of consent withdrawal, 10 because of an unsatisfactory therapeutic effect, and five because of AEs. Overall, 98.1 % (159/162) of patients who received at least one dose of pasireotide reported at least one AE, regardless of study-drug relationship, from core baseline until the 24-month data cut-off. Of these patients, 48.4 % experienced AEs with a maximum grade of 1 or 2 (based on CTCAE criteria). The most frequently observed AEs were gastrointestinal disorders, which were reported in 81.5 % of patients. The most common AEs suspected to be study drug related were diarrhea (55.6 %), nausea (48.1 %), hyperglycemia (38.9 %), and cholelithiasis (31.5 %) (Table 2); 11.7 % and 8.6 % of patients experienced grade 3 or 4 hyperglycemia and diabetes mellitus, respectively. Newly occurring AEs during the extension phase that were suspected to be drug related included cholelithiasis (n = 3), diabetes mellitus, nausea, fatigue, diarrhea (n = 2 each), headache and elevated alanine transaminase (n = 1 each). No new safety issues were identified after the first 12-month data cut-off. Serious AEs (SAEs) were reported by 25.9 % (42/162) of patients who received at least one dose of pasireotide; two patients who did not have an SAE up to the core study data cut-off subsequently experienced ≥1 SAE. There were no deaths throughout the entire study.

**Table 2:** Most frequently reported AEs suspected to be study drug related (occurring in ≥10 % of patients in either dose group) up to the 24-month data cut-off in the 162 patients who received at least one dose of pasireotide

AE, n (%)	Pasireotide 600 µg bid (N = 82)		Pasireotide 900 µg bid (N = 80)		Overall (N = 162)	
	Grades 1 and 2	All grades	Grades 1 and 2	All grades	Grades 1 and 2	All grades
Diarrhea	44 (53.7)	47 (57.3)	42 (52.5)	43 (53.8)	86 (53.1)	90 (55.6)
Nausea	34 (41.5)	35 (42.7)	40 (50.0)	43 (53.8)	74 (45.7)	78 (48.1)
Hyperglycemia	23 (28.0)	30 (36.6)	21 (26.3)	33 (41.3)	44 (27.2)	63 (38.9)
Cholelithiasis	27 (32.9)	28 (34.1)	22 (27.5)	23 (28.8)	49 (30.2)	51 (31.5)
Abdominal pain	13 (15.9)	14 (17.1)	17 (21.3)	19 (23.8)	30 (18.5)	33 (20.4)
Diabetes mellitus	9 (11.0)	17 (20.7)	10 (12.5)	16 (20.0)	19 (11.7)	33 (20.4)
Fatigue	6 (7.3)	7 (8.5)	12 (15.0)	14 (17.5)	18 (11.1)	21 (13.0)
HbA_1c_ elevation	9 (11.0)	10 (12.2)	7 (8.8)	7 (8.8)	16 (9.9)	17 (10.5)
ALT elevation	9 (11.0)	10 (12.2)	3 (3.8)	5 (6.3)	12 (7.4)	15 (9.3)
Type 2 diabetes mellitus	6 (7.3)	10 (12.2)	2 (2.5)	5 (6.3)	8 (4.9)	15 (9.3)
Headache	5 (6.1)	6 (7.3)	8 (10.0)	8 (10.0)	13 (8.0)	14 (8.6)
Vomiting	1 (1.2)	2 (2.4)	7 (8.8)	8 (10.0)	8 (4.9)	10 (6.2)

AEs are ordered based on the frequency of events. *ALT* alanine transaminase. The 24-month data cut-off for AE analysis occurred when the last ongoing patient reached 24 months’ pasireotide treatment. As such, a number of patients received >24 months’ treatment. At the 24-month data cut-off, the duration of treatment for those patients who received ≥1 dose of pasireotide ranged from 0 to 50 months (mean 14 months)

### Concomitant medication

Thirty-six patients (62.1 %) received antihypertensive medication at some stage during the core study or extension. Of the 28 patients who were not receiving antihypertensive medication at core baseline, six received ≥1 antihypertensive agent at some stage during the core study or extension; eight of the 30 patients who were receiving antihypertensive medication at core baseline received ≥1 additional agent. Thirty-five patients (60.3 %) received antidiabetic medication at some stage during the core study or extension. Of the 50 patients who were not receiving antidiabetic medication at core baseline, at least one antidiabetic medication was initiated in 27 patients; five of the eight patients who were receiving antidiabetic medication at core baseline received ≥1 additional agent during the core study or extension. Only one of 24 patients who did not receive any antidiabetic medication during the core study initiated an antidiabetic treatment during the extension phase. One additional patient had an increase in the number of antidiabetic medications they received during the extension phase compared with the core study.

### Blood glucose

**Fig. 4:**
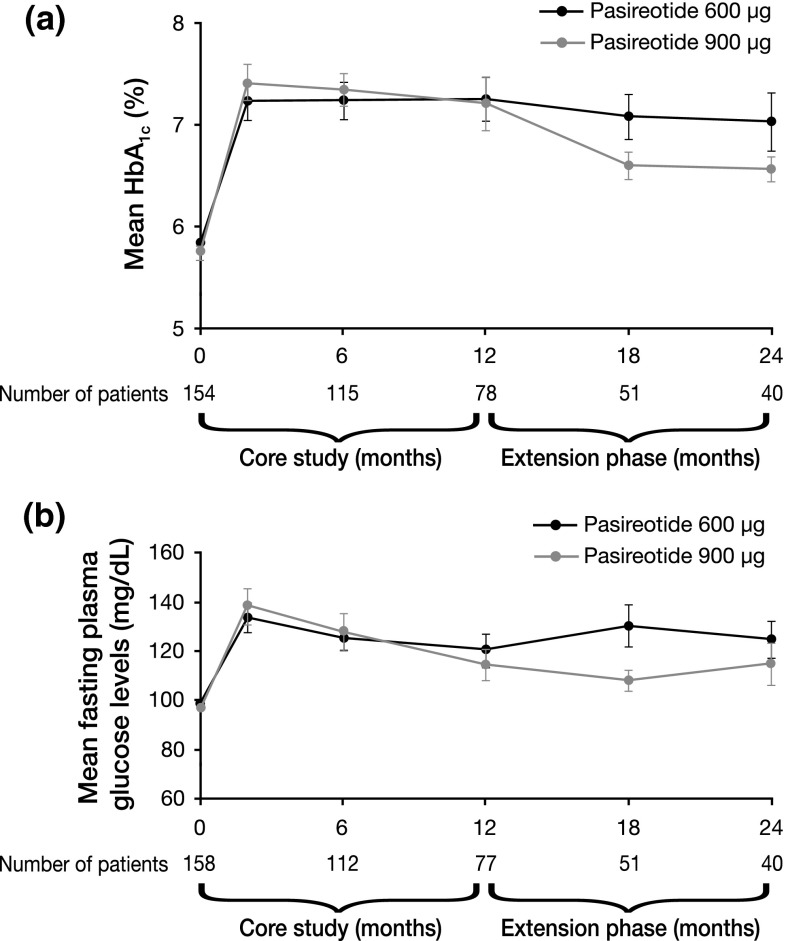
Mean (±SE) **a** HbA_1c_ and **b** fasting plasma glucose over time from baseline up to 24-month data cut-off in the 162 patients who received at least one dose of pasireotide

Mean fasting plasma glucose and HbA_1c_ levels increased soon after initiation of pasireotide therapy; peak fasting glucose levels were achieved by month 1 and stabilized thereafter. Mean HbA_1c_ increased from 5.8 % at core baseline to 7.2 % and 6.8 % following 12 and 24 months’ treatment, respectively (Fig. 4a); for the 58 patients who entered the extension, mean HbA_1c_ was similar at month 24 compared with extension baseline (6.8 vs. 7.0 %). Similarly, mean fasting plasma glucose increased from 97.8 mg/dL at core baseline to 117.7 mg/dL at month 12 and then remained stable until month 24 (119.5 mg/dL) (Fig. 4b); for the 58 patients who entered the extension, mean fasting plasma glucose was similar at month 24 compared with extension baseline (119.5 vs. 113.1 mg/dL).

For those patients who did not receive antidiabetic medication during the 24-month study period, 21 had baseline and post-baseline HbA_1c_ measurements; 61.9 % (13/21) of these patients had a last available HbA_1c_ level indicating a worse diabetic status than at core baseline. Two patients had missing values at core baseline. Further details can be found in the Supplementary Appendix.

Most patients had an increase in HbA_1c_ relative to baseline at some point after initiation of pasireotide treatment. At baseline, 49.4 % (80/162) of patients had normal HbA_1c_ levels (HbA_1c_ <5.7 %); 76.3 % (61/80) of these patients had last available HbA_1c_ levels in a category indicating a worse diabetic status than at core baseline (Table 3). Overall, 16.0 % (26/162) of patients had baseline HbA_1c_ levels in the diabetic range (HbA_1c_ ≥6.5 %). Of the patients who had baseline HbA_1c_ levels in the diabetic range and ≥1 post-baseline measurement, all had at least one post-baseline value indicating poorly controlled diabetes (HbA_1c_ levels ≥ 8 %; data not shown) and a last available value indicating diabetes mellitus or poorly controlled diabetes (Table 3).

**Table 3:** Overall shift in HbA_1c_ from baseline to last available value up to 24-month data cut-off in the 162 patients who received at least one dose of pasireotide

	Baseline	Last reported HbA_1c_ value				
		<5.7 %	5.7 to <6.5 %	6.5 to <8 %	≥8 %	Missing
	n (%)	n (%)	n (%)	n (%)	n (%)	n (%)
<5.7 %	80 (49.4)	14 (8.6)	*32 (19.8)*	*27* (*16.7*)	*2 (1.2)*	5 (3.1)
5.7 to <6.5 %	48 (29.6)	2 (1.2)	7 (4.3)	*26* (*16.0*)	*12* (*7.4*)	1 (0.6)
6.5 to <8 %	24 (14.8)	0	0	5 (3.1)	*18* (*11.1*)	1 (0.6)
≥8 %	2 (1.2)	0	0	0	2 (1.2)	0
Missing	8 (4.9)	0	3 (1.9)	4 (2.5)	0	1 (0.6)
Total	162 (100.0)	16 (9.9)	42 (25.9)	62 (38.3)	34 (21.0)	8 (4.9)

Italicized values represent the patients with a shift in HbA_1c_ level that indicates a worse diabetic status at the last reported value compared with core baseline. Two patients with HbA_1c_ ≥8 % at study entry received pasireotide treatment (one patient in each treatment arm) and were classed as protocol deviations

## Discussion

This long-term study reports the efficacy and safety of pasireotide sc following 24 months of treatment in patients with de novo, persistent or recurrent Cushing’s disease [9]. Initiation of pasireotide treatment resulted in rapid and sustained mean reductions in UFC, serum cortisol and ACTH levels, which were accompanied by persistent improvements in clinical signs of Cushing’s disease. Pasireotide was generally well tolerated over the 24-month treatment period.

In the 58 patients who completed the core study and entered the extension, the mean percentage decrease in UFC level from baseline following 12 and 24 months of pasireotide treatment was 57–62 % and was similar in both dose groups. There was an overall decrease in the proportion of patients with controlled UFC at month 24 compared with month 12; 50.0 % (29/58) and 34.5 % (20/58) of patients had controlled UFC at month 12 and month 24, respectively. As patients without UFC values were considered to be uncontrolled, the lack of UFC values at month 24 for three patients (two patients had discontinued and one patient had a missing UFC value) who had controlled UFC at their last available assessment as well as at month 12 may have contributed to the observed decrease in number of responders between months 12 and 24. While escape from response has been reported during long-term treatment of Cushing’s disease with the dopamine receptor agonist cabergoline [14, 15], a similar phenomenon has not been shown for pasireotide. Nevertheless, it is possible that a loss of pasireotide efficacy in a small number of patients may have contributed to the reduction in the number of responders during the extension study.

Reductions in UFC with pasireotide treatment were accompanied by rapid decreases in mean serum cortisol and plasma ACTH levels, which were initially observed in the core study and were maintained for the duration of the extension (serum cortisol and plasma ACTH levels decreased by 18.0 and 14.7 %, respectively, from core baseline to month 24). Sustained improvements in a number of clinical signs, including systolic and diastolic blood pressure, weight, and total cholesterol level, were also observed; changes in signs and symptoms during 12 months of treatment with pasireotide have been recently reported [16]. The data from the current 24-month study suggest that the initial efficacy and clinical benefit of pasireotide in patients with Cushing’s disease can be maintained over an extended 24-month time period. However, as changes in the dose of concomitant medications were not recorded during the 24-month treatment period, further studies are necessary to determine the effects of these therapies on the observed improvements in clinical signs and symptoms. Interestingly, a subanalysis of patients with hypertension at core baseline showed that 62.5 % (10/16) of those patients who did not receive antihypertensive drugs during the core study had a >5 mmHg decrease in diastolic blood pressure after 12 months of pasireotide treatment [17].

Pasireotide showed a similar safety profile to that of other somatostatin analogues, except for the increased frequency and degree of hyperglycemia. The safety profile of pasireotide following 24 months’ treatment was similar to that reported at 12 months, with no new safety issues identified during the extension.

The development of hyperglycemia is a well-characterized effect of pasireotide treatment [18]. Plasma glucose and HbA_1c_ levels increased soon after initiation of pasireotide but did not deteriorate further over 2 years of treatment. The stabilization of plasma glucose and HbA_1c_ levels over time may have resulted from a number of factors, including the administration of antidiabetic medication, correction of hypercortisolism, and patient discontinuations. However, these results should be interpreted with caution because of the smaller number of patients who entered the extension phase compared with the 12-month core study. Recent studies in healthy volunteers showed that hyperglycemia associated with pasireotide treatment results from a decrease in incretin and insulin secretion, with no change in insulin sensitivity [18]. Patients on pasireotide treatment should be closely monitored for changes in glucose homeostasis, and prompt intervention is warranted if hyperglycemia occurs.

One limitation of this study is that all the efficacy data are derived from the 58 patients who entered the extension based on the achievement of controlled UFC levels or significant clinical benefit during the core study. As such, it could be considered that these patients were ‘pre-selected’ for a positive response to pasireotide. Nevertheless, these data are encouraging and suggest that patients who have a biochemical response to pasireotide respond rapidly (during the first 2 months of treatment) and can continue to respond over a sustained period of time. Non-responders could therefore be identified soon after initiating pasireotide treatment and treatment decisions made early in the therapeutic course, taking into account both biochemical and clinical response. Of note, the baseline characteristics of the patients entering the extension were similar to those of the overall core patient population, with no obvious characteristic predictive of a positive response to pasireotide treatment.

## Conclusions

Long-term use of pasireotide resulted in sustained improvements in mean UFC, serum cortisol and plasma ACTH. Significant improvements in blood pressure, weight and total cholesterol were also maintained for the duration of treatment. The safety profile of pasireotide was typical for a somatostatin analogue, except for changes in glucose homeostasis, which need to be closely monitored in patients receiving pasireotide treatment. This study shows that pasireotide provides sustained clinical benefit, supporting its use as a treatment for patients with Cushing’s disease.

## Electronic supplementary material

Below is the link to the electronic supplementary material.

Supplementary material 1 (DOCX 22 kb)

## References

[1] Newell-Price J, Bertagna X, Grossman AB, Nieman LK (2006). Cushing’s syndrome. Lancet.

[2] Biller BMK, Grossman AB, Stewart PM, Melmed S, Bertagna X, Bertherat J, Buchfelder M, Colao A, Hermus AR, Hofland LJ, Klibanski A, Lacroix A, Lindsay JR, Newell-Price J, Nieman LK, Petersenn S, Sonino N, Stalla GK, Swearingen B, Vance ML, Wass JA, Boscaro M (2008). Treatment of adrenocorticotropin-dependent Cushing’s syndrome: a consensus statement. J Clin Endocrinol Metab.

[3] Pivonello R, De Martino MC, De Leo M, Lombardi G, Colao A (2008). Cushing’s syndrome. Endocrinol Metab Clin North Am.

[4] Data from CDC statistics and NHANES III (2005–2006). 2011. Available at: http://www.cdc.gov/nchs/nhanes.htm

[5] Feelders RA, Pulgar SJ, Kempel A, Pereira AM (2012). The burden of Cushing’s disease: clinical and health-related quality of life aspects. Eur J Endocrinol.

[6] Bruns C, Lewis I, Briner U, Meno-Tetang G, Weckbecker G (2002). SOM230: a novel somatostatin peptidomimetic with broad somatotropin release inhibiting factor (SRIF) receptor binding and a unique antisecretory profile. Eur J Endocrinol.

[7] Batista DL, Zhang X, Zhou Y, Ansell P, Gejman R, Stratakis CA, Swearingen B, Johnson SA, Kilbanski A (2006) SOM230 inhibits cell proliferation in human corticotroph adenomas and in human pituitary tumor-derived PDFS cell line. 88th Annual Meeting of the Endocrine Society. Boston, MA, USA, 24–27 June:abst P2-755

[8] Hofland LJ, Van Der Hoek J, Feelders R, van Aken MO, van Koetsveld PM, Waaijers M, Sprij-Mooij D, Bruns C, Weckbecker G, de Herder WW, Beckers A, Lamberts SW (2005). The multi-ligand somatostatin analogue SOM230 inhibits ACTH secretion by cultured human corticotroph adenomas via somatostatin receptor type 5. Eur J Endocrinol.

[9] Colao A, Petersenn S, Newell-Price J, Findling JW, Gu F, Maldonado M, Schoenherr U, Mills D, Salgado LR, Biller BMK (2012). A 12-month Phase 3 study of pasireotide in Cushing’s disease. N Engl J Med.

[10] Novartis Pharma AG (2012) Signifor summary of product characteristics. Available at: http://www.signifor.com/european-product-characteristics.jsp (last accessed February 2013)

[11] Novartis Pharma AG (2012) Signifor full prescribing information. Available at: http://www.pharma.us.novartis.com/cs/www.pharma.us.novartis.com/product/pi/pdf/signifor.pdf

[12] Turpeinen U, Markkanen H, Valimaki M, Stenman UH (1997). Determination of urinary free cortisol by HPLC. Clin Chem.

[13] National Cancer Institute (2006) Common terminology criteria for adverse events v3.0 (CTCAE). Available at: http://ctep.cancer.gov/protocolDevelopment/electronic_applications/docs/ctcaev3.pdf

[14] Godbout A, Manavela MP, Danilowicz K, Beauregard H, Bruno OD, Lacroix A (2010). Cabergoline monotherapy in the long-term treatment of Cushing’s disease. Eur J Endocrinol.

[15] Pivonello R, De Martino MC, Cappabianca P, De Leo M, Faggiano A, Lombardi G, Hofland LJ, Lamberts SW, Colao A (2009). The medical treatment of Cushing’s disease: effectiveness of chronic treatment with the dopamine agonist cabergoline in patients unsuccessfully treated by surgery. J Clin Endocrinol Metab.

[16] Pivonello R, Petersenn S, Newell-Price J, Findling J, Gu F, Maldonado M, Trovato A, Hughes G, Salgado L, Lacroix A, Schopohl J, Biller B (2014). Pasireotide treatment significantly improves clinical signs and symptoms in patients with Cushing’s disease: results from a Phase III study. Clin Endocrinol (Oxf).

[17] Pivonello R, Petersenn S, Newell-Price J, Gu F, Maldonado M, Trovato A, Hughes G, Salgado LR, Lacroix A, Schopohl J, Biller BM (2012). Pasireotide treatment is associated with improvements in hypertension: 12-month results from a large Phase III study in Cushing’s disease. Endocr Abstr.

[18] Henry RR, Ciaraldi TP, Armstrong D, Burke P, Ligueros-Saylan M, Mudaliar S (2013). Hyperglycemia associated with pasireotide: results from a mechanistic study in healthy volunteers. J Clin Endocrinol Metab.

