# Supplementary material for: Pasireotide can induce sustained decreases in urinary cortisol and provide clinical benefit in patients with Cushing’s disease: results from an open-ended, open-label extension trial
Source: Pituitary. 2014 Dec 24;18(5):604–12. doi: 10.1007/s11102-014-0618-1 (PMC4560758; doi:10.1007/s11102-014-0618-1)
Supplement: Supplementary file 1 — Supplementary material 1 (DOCX 22 kb) [file 11102_2014_618_MOESM1_ESM.docx]

**Supplementary Appendix**

**Pasireotide can induce sustained decreases in urinary cortisol and provide clinical benefit in patients with Cushing’s disease: results from an open-ended, open-label extension trial**

Schopohl J *et al*.

Overall shift in HbA_1c_ from baseline to last available value up to 24-month data cut-off for the 23 patients who did not receive antidiabetic medication during the 24-month study period

|  | **Baseline** | | **Last reported HbA_1c_ value** | | | | |
| --- | --- | --- | --- | --- | --- | --- | --- |
|  | |  | **<5.7%** | **5.7% to <6.5%** | **6.5% to <8%** | **≥8%** | **Missing** |
|  | | **n (%)** | **n (%)** | **n (%)** | **n (%)** | **n (%)** | **n (%)** |
| <5.7% | | 16 (69.6) | 6 (26.1) | 3 (13.0) | 7 (30.4) | 0 | 0 |
| 5.7% to <6.5% | | 5 (21.7) | 0 | 2 (8.7) | 3 (13.0) | 0 | 0 |
| 6.5% to <8% | | 0 | 0 | 0 | 0 | 0 | 0 |
| ≥8% | | 0 | 0 | 0 | 0 | 0 | 0 |
| Missing | | 2 (8.7) | 0 | 2 (8.7) | 0 | 0 | 0 |
| Total | | 23 (100.0) | 6 (26.1) | 7 (30.4) | 10 (43.5) | 0 | 0 |

Shaded boxes represent the patients with a shift in HbA_1c_ level that indicates a worse diabetic status at the last reported value compared with core baseline.

**Pasireotide B2305 Study Group**

Members of the Pasireotide B2305 Study Group included: R Abs, B Allolio, R Auchus,
J Bertherat, BMK Biller, C Boguszewski, M Boscaro, T Brue, O Bruno, P Caron,
F Cavagnini, D Carvalho, O Chabre, A Chervin, C Chik, A Colao, A Çömlekçi, C Cortet, M Czepielewski, E Degli Uberti, A Estour, U Feldt-Rasmussen, M Fleseriu, P Freda,
M Gadelha, M Gershinsky, E Ghigo, B Glaser, F Gu, M Guitelman, S Guler,
A Hamrahian, SA Imran, Z Jin, JO Jorgensen, P Kadıoğlu, L Katznelson, A Lacroix,
A Leal Cerro, P Loli, L Lu, W Ludlam, F Mantero, E Martino, M Mercado, A Moreira,
K Nogueira, G Ning, L Østergaard Kristensen, S Petersenn, G Piaditis, L Portocarrero,
V Rohmer, R Rubens, L Salgado, S Samson, J Schopohl, I Shimon, C Strasburger,
A Tabarin, M Terzolo, S Tsagarakis, N Unger, Z Valkusz, L Van Gaal, M Välimäki,
E Vidal, S Webb, R Weiss, W Zgliczyński.
